# A Rare Case of Glossitis due to* Pasteurella multocida* after a Cat Scratch

**DOI:** 10.1155/2016/2868505

**Published:** 2016-10-20

**Authors:** Negin Niknam, Thien Doan, Elizabeth Revere

**Affiliations:** Hofstra Northwell School of Medicine, Hempstead, NY, USA

## Abstract

*Pasteurella* is one of the zoonotic pathogens that can cause variety of serious infections in animals and humans such as bacteremia, septic shock, endocarditis, meningitis, prosthetic and native valve infections, osteomyelitis, skin and soft tissue infections, abscesses, and even pneumonia with empyema. However, there have been few reports of upper respiratory involvements like tonsillitis and epiglottitis in humans. We present a case of recurrent* Pasteurella* glossitis after a cat scratch which has not been reported in humans.

## 1. Introduction

*Pasteurella* are small Gram-negative coccobacilli that are primarily animal pathogens.* Pasteurella multocida* is a component of the normal upper respiratory tract flora of fowl and mammals, especially felines. Other* Pasteurella* species can be found in the oral cavity of a variety of animals including dogs, cats, pigs, hamsters, and horses.

*Pasteurella* can cause a variety of diseases in animals, such as fowl cholera in domestic fowl, shipping fever in cattle, hemorrhagic septicemia in cattle and lambs, fibrinous pneumonia in cattle, snuffles in rabbits, and other focal infections [1]. However these organisms can cause several infections in humans, usually as a result of cat scratches or cat or dog bites or licks. There have been multiple cases of septic shock [2, 3], bacteremia [4], prosthetic valve endocarditis [5, 6], aortic endograft infection [7], primary shoulder involvement [8], total knee replacement infection [9], meningitis [10, 11], and even pneumonia [12, 13] or empyema [14]. It was also reported in deep sternal wound infection [15], peritonitis due to peritoneal dialysis [16, 17], endophthalmitis [18], chorioamnionitis from vaginal transmission [19], frontal osteomyelitis Pott's puffy tumor [20], infection in solid organ transplantation [21], lung and liver abscess [22], and even failed renal transplant [23]. However, there were rare cases of tonsillitis [24] or epiglottitis [25, 26] in humans. There has been one report of Ludwig's angina after a dog bite [27] but glossitis was mostly seen in animals [28].

## 2. Case Presentation

A 56-year-old male with a past medical history of hypertension, hyperlipidemia, gastroesophageal reflux disease, recurrent otitis media leading to hearing impairment, and tonsillectomy, presented to the Emergency Department with tongue swelling, dysphagia, drooling, and shortness of breath. He stated that about 5 days prior to presentation he experienced throat pain and went to his Ear, Nose, Throat (ENT) specialist, who felt his symptoms could be due to lymphadenitis. He was prescribed Clindamycin, which the patient had tolerated in the past. He continued to have worsening throat pain and difficulty swallowing and presented to the Emergency Department. In the Emergency Department a CT scan of his neck was performed and was unremarkable as well as direct laryngoscopy. He was discharged with Dexamethasone and Ketorolac. The next day he started to develop severe tongue pain and swelling to the point that he could not speak or move his tongue. He was evaluated by ENT specialist who noticed tongue edema tenderness and enlargement suggestive of glossitis with no posterior pharyngeal edema.

His laboratory work shown was remarkable for a white blood cell count: 14.1 K/uL, neutrophil count: 11.73 K/uL, hemoglobin: 15.2 g/dL, and platelets count: 177 K/uL.

A repeat CT scan of the neck with contrast showed asymmetric fatty reticulation along the left sublingual space without focal mass or rim-enhancing fluid collection and no evidence of odontogenic abscess. Blood cultures were sent and the patient was admitted to the ICU for glossitis or possible Ludwig's angina. He was initially started on Dexamethasone and Clindamycin in view of his penicillin allergy which was remote and reported as a rash.

On the second day, blood cultures revealed Gram-negative rods. The antibiotics were switched from Clindamycin to Aztreonam and Metronidazole. The next day the Gram-negative organism was identified as* Pasteurella multocida*. On further investigation, the patient stated that he was scratched by his new cat two weeks prior on his right wrist, which resolved without complications. Aztreonam and Metronidazole were discontinued and the patient was started on Imipenem.

Further laboratory work showed improving leukocytosis and repeat blood cultures were negative the next day. Echocardiogram was performed and showed normal valves and normal right ventricular size and function, but moderate to severe global left ventricular dysfunction. The patient was diagnosed with systolic heart failure. The patient's symptoms gradually resolved and he was discharged home with Ertapenem via PICC line for a total of 14 days. However, 10 days after discharge the patient presented again with a milder swelling of his tongue with no signs of sepsis or airway compromise. He was admitted again and blood cultures were sent which were negative. The white blood cell count and neck and chest X-rays were within normal limits. The course of Ertapenem was completed and he was discharged home. The patient did not report any further episodes of tongue or throat swelling.

## 3. Discussion

*P. multocida* can cause a range of infections in wild and domesticated animals as well as humans from a mild wound infection to severe sepsis and death. The diseases are usually different in animals, such as the glossitis that was reported only in animals.

Infections with* P. multocida* can be divided into three categories in humans:

- Skin and soft tissue infections: following animal bites or scratches; bites and scratches can also result in abscesses, necrotizing soft tissue infections, septic arthritis, and osteomyelitis.
- Serious invasive infection often unrelated to animal bites, such as meningitis, intraabdominal infection, endocarditis, or ocular infection.
- Oral and respiratory infections, usually in the setting of chronic pulmonary disease.

*Pasteurella* respiratory infections are rare and have no distinctive characteristics.* P. multocida* may not be suspected as the infecting pathogen but should be considered as a serious pathogen in patients with cat exposure including bites, licks, and scratches.

Glossitis has been never reported in humans and is not well described in the literature. In our case diagnosis was made clinically.

## 4. Conclusion

Glossitis could be a rare presentation of* Pasteurella multocida*, which can be diagnosed clinically and treated the same as other* Pasteurella* infections.
